# High-resolution phenomics dataset collected on a field-grown, EMS-mutagenized sorghum population evaluated in hot, arid conditions

**DOI:** 10.1186/s13104-025-07407-9

**Published:** 2025-07-29

**Authors:** Jeffrey Demieville, Brian Dilkes, Andrea L. Eveland, Duke Pauli

**Affiliations:** 1 School of Plant Sciences, The University of Arizona, Tucson, AZ 85721 United States of America; 2 Department of Biochemistry, Purdue University, West Lafayette, IN 47907 United States of America; 3 Donald Danforth Plant Science Center, St. Louis, MO 63132 United States of America; 4 Center for Agroecosystem Research in the Desert (ARID), The University of Arizona, Tucson, AZ 85721 United States of America

**Keywords:** Sorghum, High performance computing, Field phenotyping, Drought, EMS mutagenesis, Thermal, RGB, PSII, 3D topological data analysis, Arizona

## Abstract

**Objectives:**

The University of Arizona Field Scanner (FS) is capable of generating massive amounts of data from a variety of instruments at high spatial and temporal resolution. The accompanying field infrastructure beneath the system offers capacity for controlled irrigation regimes in a hot, arid environment. Approximately 194 terabytes of raw and processed phenotypic image data were generated over two growing seasons (2020 and 2022) on a population of 434 sequence-indexed, EMS-mutagenized sorghum lines in the genetic background BTx623; the population was grown under well-watered and water-limited conditions. Collectively, these data enable links between genotype and dynamic, drought-responsive phenotypes, which can accelerate crop improvement efforts. However, analysis of these data can be challenging for researchers without background knowledge of the system and preliminary processing.

**Data description:**

This dataset contains formatted tabular data generated from sensing system outputs suitable for a wide range of end-users and includes plant-level bounding areas, temperatures, and point cloud characteristics, as well as plot-level photosynthetic parameters and accompanying weather data. The dataset includes approximately 422 megabytes of tabular data totaling 1,903,412 unique unfiltered rows of FS data, 526,917 cleaned rows of FS data, and 285 rows of weather data from the two field seasons.

## Objective

Sorghum has potential as a high-yielding bioenergy crop because of its resilience to drought and heat stresses that make it well-suited for production in challenging environments. However, biomass yield is still limited by these stresses. Identifying the genes and pathways that function in sorghum’s responses to water deficit will help accelerate the development of improved biomass varieties. To provide the community with a resource for linking genotype to drought-responsive phenotypes observed under field conditions, a population of 434 ethyl methanesulfonate (EMS)-mutagenized M5 sorghum lines (BTx623 background) that have been completely sequenced [1, 2] were extensively phenotyped over two growing seasons (2020 and 2022). The University of Arizona Field Scanner was used to collect high spatial and temporal resolution field phenomics data with a variety of sensors on this population grown under controlled irrigation conditions in both years. Processed data from these various sensors can be integrated to generate hypotheses on morph-physiological responses of sorghum over the course of a growing season and in response to abiotic stresses, and how sequence mutations drive phenotypic outputs. Approximately 194 terabytes of raw and processed phenotypic image data were generated and made publicly accessible. Through analysis of these data, researchers can leverage information on dynamic phenotypic traits to study genotype-to-phenotype associations [3] in response to drought and derive testable hypotheses on gene function. These multifeatured data can also be used in advanced modeling [4–6] and AI-driven predictions of plant growth and phenotypic responses to water deficit conditions with reduced costs. Generating this dataset relied on extensive knowledge of instrument capabilities and limitations, FS operational paradigms, and project data engineering and processing pipelines, as well as high-performance computing and cyberinfrastructure resources. This dataset was created to serve as a product that is ready for exploration by end-users, providing all information necessary to conduct further analysis.

## Data description

Data were collected from two sorghum field trials grown at the University of Arizona’s Maricopa Agricultural Center (33°04’24.8” N 111°58’25.7” W, elevation 366 m) in 2020 and 2022. A population of 434 sequence-indexed, EMS-mutagenized sorghum lines in the BTx623 genetic background was phenotyped over both growing seasons using the University of Arizona Field Scanner, a robotic high-throughput phenotyping platform carrying a suite of cameras and sensors [7]. Field trials were arranged in a randomized incomplete block design with partial replication per irrigation treatment. Irrigation treatment levels, representing well-watered (WW, field capacity) and water-limited (WL, 60% of WW) conditions, were managed using subsurface drip irrigation, and conducted by splitting the field into two halves. The irrigation treatment was imposed approximately 50 days post emergence. Plots were hand-seeded at 20 seeds each and, after plant establishment, thinned to five plants each to enable individual plant phenotyping. Standard cultivation practices were employed for crop management.

The dataset consists of FS tabular data generated from red-green-blue (RGB), forward-looking thermal (FLIR), and photosystem II (PSII) chlorophyll fluorescence imagers, FS tabular data generated from 3D point clouds captured by structured light 3D scanners, tabular weather data collected by the Arizona Meteorological Network (AZMET) [8] Maricopa station, and associated experimental design and genotypic identifying information. Each type of data was collected at several time points over both growing seasons to capture dynamic growth and water limitation responses. Raw FS data was transferred to CyVerse and processed using PhytoOracle [9], a series of scalable, modular phenomic data processing pipelines. Outputs include plant-level bounding areas (RGB, FLIR), canopy and region of interest temperatures (FLIR), point cloud descriptive metrics and topological data analysis features (3D) extracted using the Giotto-TDA package [10], and plot-level photosynthetic parameters (PSII). Plant-level metrics are associated across RGB, FLIR, and 3D data with common individual plant identifications and are available through a CyVerse dashboard [11, 12]. Unique accession identifiers are included for each sorghum line traceable to full sequence information including variant calls through SorghumBase [13] and to USDA-ARS Germplasm Resources Information Network (GRIN) [14] germplasm stock.

The dataset [15] combines data collected by various sensing systems at different timepoints with experimental design to result in the *2020_full* and *2022_full* datasets. From these datasets, RGB and FLIR data were initially filtered, removing individual plants not detected on at least 67% of days. 3D data were initially filtered, removing individual plants with num_points below the 90th percentile. Outliers were removed as quantified by Tukey fences (*k = 1.5*) on traits bounding_area_m2 (RGB, FLIR), roi_temp (FLIR), canopy_temp_mean (FLIR), canopy_temp_median (FLIR), num_points (3D), hull_volume (3D), and FV/FM (PSII), resulting in *2020_cleaned* and *2022_cleaned*. AZMET data is included in *weather*. Header descriptions are captured in *header_information*. Supplementary information is captured in *ReadMe*.

The dataset summarizes approximately 194 terabytes (TB) of image data to approximately 422 megabytes (MB) of tabular data, including the following, as noted in Table 1:

- 2020_full: 660,433 rows.
- 2020_cleaned: 293,646 rows.
- 2022_full: 1,242,979 rows.
- 2022_cleaned: 233,271 rows.
- weather: 285 rows.

**Table 1:** Overview of data files/data sets

Label	Name of data file/data set	File types (file extension)	Data repository and identifier (DOI or accession number)
*Data file 1*	*2020_full*	.*zip (folder with.csv files)*	CyVerse Data Commons (10.25739/zpnk-9v06) [15]
*Data file 2*	*2020_cleaned*	.*zip (folder with.csv files)*	CyVerse Data Commons (10.25739/zpnk-9v06) [15]
*Data file 3*	*2022_full*	.*zip (folder with.csv files)*	CyVerse Data Commons (10.25739/zpnk-9v06) [15]
*Data file 4*	*2022_cleaned*	.*zip (folder with.csv files)*	CyVerse Data Commons (10.25739/zpnk-9v06) [15]
*Data file 5*	*weather*	.*zip (folder with.csv files)*	CyVerse Data Commons (10.25739/zpnk-9v06) [15]
*Data file 6*	*header_information*	.*tsv*	CyVerse Data Commons (10.25739/zpnk-9v06) [15]
*Data file 7*	*ReadMe*	.*txt*	CyVerse Data Commons (10.25739/zpnk-9v06) [15]

### Limitations

Field-based research and unique equipment like the FS are challenged in collecting continuous high-quality field phenomic data. Ambient conditions, hardware complexity, software issues, and instrument limitations all affect the output data.

- The field environment for the experiment presented numerous challenges. At times, the extreme heat of the environment was greater than instrument cooling capability, potentially affecting quality of the thermal data. Variable light conditions during a scan mission resulted in over- and under-exposed images. High winds affect the stability of the FS and introduce movement to both the FS machine and the sorghum plants, affecting 3D point clouds.
- FS software lacked an effective method for performing real-time quality control of data. This limited the ability to make on-the-fly adjustments to camera settings for maximum quality. Over- and under-exposed images were present at some stages of processing.
- Ability to capture quality 3D point clouds was affected by drastic height differences between neighboring genotypes due to occlusion and depth of field limitations. Scan missions were targeted at the mode height of the field and adjusted as plant height increased. As a result, when plants were significantly shorter or taller than the targeted height, they may have been missed during the acquisition.
- Removal of outliers in the cleaned dataset may result in discarding information of interest. Genotypic variation, that would otherwise be missed, may be captured by those outliers. Cleaned data in the dataset discards point clouds below the 90th percentile for the number of points. As a result, good data corresponding to severely growth-inhibited plants may be discarded during cleaning.

## Data Availability

The data described in this Data note can be freely and openly accessed on CyVerse Data Commons under DOI 10.25739/zpnk-9v06. Please see Table 1 and references [15] for details and links to the data.

## Abbreviations

FS: Field Scanner
EMS: Ethyl methanesulfonate
WW: Well-watered
WL: Water-limited
RGB: Red, green, blue
FLIR: Forward-looking infrared
PSII: Photosystem II
3D: Three-dimensional
AZMET: Arizona Meteorological Network
GRIN: Germplasm Resources Information Network
